# Gut Microbiota Dysbiosis Influences Metabolic Homeostasis in *Spodoptera frugiperda*

**DOI:** 10.3389/fmicb.2021.727434

**Published:** 2021-09-30

**Authors:** Yaqing Chen, Huanchan Zhou, Yushan Lai, Qi Chen, Xiao-Qiang Yu, Xiaoyun Wang

**Affiliations:** Guangdong Provincial Key Laboratory of Insect Developmental Biology and Applied Technology, School of Life Sciences, Institute of Insect Science and Technology, South China Normal University, Guangzhou, China

**Keywords:** gut microbiota, antibiotics, metabolic homeostasis, energy, autophagy, *Spodoptera frugiperda*

## Abstract

Insect gut microbiota plays important roles in acquiring nutrition, preventing pathogens infection, modulating immune responses, and communicating with environment. Gut microbiota can be affected by external factors such as foods and antibiotics. *Spodoptera frugiperda* (Lepidoptera: Noctuidae) is an important destructive pest of grain crops worldwide. The function of gut microbiota in *S. frugiperda* remains to be investigated. In this study, we fed *S. frugiperda* larvae with artificial diet with antibiotic mixture (penicillin, gentamicin, rifampicin, and streptomycin) to perturb gut microbiota, and then examined the effect of gut microbiota dysbiosis on *S. frugiperda* gene expression by RNA sequencing. Firmicutes, Proteobacteria, Bacteroidetes, and Actinobacteria were the most dominant phyla in *S. frugiperda*. We found that the composition and diversity of gut bacterial community were changed in *S. frugiperda* after antibiotics treatment. Firmicutes was decreased, and abundance of *Enterococcus* and *Weissella* genera was dramatically reduced. Transcriptome analysis showed that 1,394 differentially expressed transcripts (DETs) were found between the control and antibiotics-treated group. The Gene Ontology (GO) and Kyoto Encyclopedia of Genes and Genomes (KEGG) results showed that antibiotics-induced dysbiosis affected many biological processes, such as energy production, metabolism, and the autophagy–lysosome signal pathway. Our results indicated that dysbiosis of gut microbiota by antibiotics exposure affects energy and metabolic homeostasis in *S. frugiperda*, which help better understand the role of gut microbiota in insects.

## Introduction

Gut microbes play important roles in insect growth, development, and many physiological processes of the individual to maintain host health through regulating gene expression (Raymann et al., 2017; Schoeler and Caesar, 2019; Zhou et al., 2021). It has been shown that gut microbes are important in absorbing nutrition, protection from parasites and pathogens, modulation of immune responses, and communications with environment (Engel and Moran, 2013; Jang and Kikuchi, 2020). The dysbiosis of intestinal microbes can affect the metabolic process. In *Drosophila*, we have demonstrated the impact of microbiome and microbiota-derived sodium butyrate on host transcriptome and metabolome with multi-omics data (Zhou et al., 2021). Our studies revealed that the changes in composition and diversity of insect’s gut microbial community can influence important physiological functions in the host through regulating gene expression.

Gut microbes can regulate herbivore-induced defenses and enhance the insect adaptation to plants (Acevedo et al., 2017; Ugwu et al., 2020), which is important for insect survival and may provide key information for pest control. Gut microbial community is often influenced by weather, temperature, diet, and other environmental factors such as antibiotics (Schoeler and Caesar, 2019; Zhang et al., 2020). In social insects like the honeybee (*Apis mellifera*), the dysbiosis of gut microbial community caused by antibiotic exposure affects bee health and elevates mortality, in part due to increased susceptibility to pathogens (Powell et al., 2014; Raymann et al., 2017), whereas in the Chinese bumblebees (genus *Bombus* comprising eight species in Zhang’s paper), antibiotics can cause changes of the sugar metabolism and distributions of antibiotic-resistant genes in main bacterial symbiont *Gilliamella* (Zhang et al., 2021). In the gypsy moth *Lymantria dispar* (Lepidoptera: Lymantriidae), gut microbiota in the larval stage eliminated by antibiotics exposure abolished *Bacillus thuringiensis* insecticidal activity and influenced the immune responses (Broderick et al., 2006). In the taro caterpillar (*Spodoptera litura*, Lepidoptera: Noctuidae), it was found that artificial diet supplemented with antibiotic streptomycin sulfate affected gut microbial diversity; the activity of digestive enzymes was significantly increased while the activity of detoxifying enzymes was significantly decreased (Thakur et al., 2016). In the diamondback moth *Plutella xylostella* (Lepidoptera: Plutellidae), antibiotics exposure was related to reduced larval growth, high mortality, malformation of the prepupae, hindered pupation, and adult emergence (Lin et al., 2015).

Gut microbiota is also related to the metabolism, immune responses, and host defense against pathogens (Engel et al., 2012; Rozadilla et al., 2020). It was reported that the mortality caused by *B. thuringiensis* treatment was reduced when compared with the control (Broderick et al., 2006). In *B. thuringiensis-*infected *Helicoverpa armigera*, the mortality of larvae was only 10% when the gut microbiota was eliminated by the 250 and 500 μg/ml antibiotics cocktail (gentamicin, penicillin, rifampicin, and streptomycin), while the mortality of larvae fed on diets devoid of antibiotics was 83.33%. These results implied that the gut microbial community is related to larval death caused by *B. thuringiensis* treatment (Paramasiva et al., 2014).

*Spodoptera frugiperda* (Lepidoptera: Noctuidae), which is native in North and South America, has rapidly invaded African and China in recent years, and is an important destructive pest of grain crops worldwide (Goergen et al., 2016; Wu et al., 2019; Li X. et al., 2020). Due to the limited studies on the function of gut microbiota in *S. frugiperda*, it is urgent to investigate the role of gut microbiota in the growth and development of *S. frugiperda*, which can provide fundamental information for improving the efficiency of insecticides influenced by the insect gut microbiota. In this study, we characterized *S. frugiperda* larvae (3-day-old sixth instar larvae, L6D3) microbiota before and after exposure to antibiotic cocktail (gentamicin, penicillin, rifampicin, and streptomycin) by 16S ribosomal RNA (rRNA) amplicon high-throughput sequencing, and then further analyzed the influences of the microbial flora on the expression of host genes in the gut by RNA sequencing (RNA-seq). Our results showed that the diversity of gut microbiota was significantly reduced by antibiotics treatment, and the genes related to metabolism process were obviously affected.

## Materials and Methods

### Insects

The *Spodoptera frugiperda* larvae were purchased from Keyun Biocontrol Engineering Co., Ltd. (Jiyuan city) in China. The larvae were fed on artificial diet consisting of soybean powder, wheat germ, and yeast as described previously (Jia et al., 2009). Insects were reared at our laboratory for three generations at 27 ± 1°C with 70 ± 5% relative humidity, and the light:dark = 16 h:8 h photoperiod.

### Antibiotics Exposure

The fourth generation of *S. frugiperda* larvae reared in our laboratory was selected for studying the effects of antibiotics on the gut microbiota of *S. frugiperda*. The newly hatched larvae were fed on artificial diet containing four antibiotics mixtures (consisting of 500 μg/ml each of penicillin, gentamicin, rifampicin, and streptomycin by dissolving 500 mg each antibiotic in 1 L artificial diet) for 14 days (from newly hatched larvae to 3-day-old sixth instar); the control larvae were fed on artificial diet without antibiotics.

### Midgut Tissue Collection

Three-day-old sixth instar larvae (L6D3) surface was sterilized with 75% ethyl alcohol for 1 min and rinsed three times with sterile water. The midguts were dissected in sterile phosphate buffer saline (PBS, pH 7.4, 140 mmol/L NaCl, 2.7 mmol/L KCl, 10 mmol/L Na_2_HPO_4_, and 1.8 mmol/L KH_2_PO_4_) (Thakur et al., 2016). Each sample contained midguts from five individual larvae, and a total of three biological replicates were performed for each treatment (control and antibiotics treatment).

### DNA Extraction and Polymerase Chain Reactions (PCR)

Bacterial genomic DNA was extracted from the midgut contents by using the classical Phenol/Chloroform/Proteinase K method (Bargues et al., 2007). The profiles of gut microbiota were detected using universal primers (forward: 5′-AGAGTTTGATCCTGGCTCAG-3′, and reverse: 5′-AAGGAGGTGATCCAGCCGCA-3′) to amplify the V3–V4 region. The PCR reactions were carried out with 10 μl of 2 × Taq PCR mixture (Biodragon, Beijing, China), 0.4 μl each of specific forward and reverse primers (10 μM), and 10 ng of genomic DNA from control or antibiotics-treated samples. The cycling parameters were 94°C for 2 min, 94°C for 30 s, and followed by 40 cycles of 60°C for 30 s and 72°C for 2 min. The PCR products were analyzed by electrophoresis on 1% agarose.

### Quantitative Real-Time PCR

Quantitative real-time PCR (qRT-PCR) reactions were performed in a total reaction volume of 25 μl, including 12.5 μl of 2 × Hieff^®^ qPCR SYBR Green Master Mix (Low Rox Plus) (YEASEN, Shanghai, China) and 0.5 μl each of specific forward and reverse primers (10 μM). The cycling parameters were 95°C for 5 min, followed by 40 cycles of 95°C for 10 s and 60°C for 30 s. The GAPDH (glyceraldehyde-3-phosphate dehydrogenase, GenBank ID: 118271716) gene was used as a reference gene. The fold changes of genes were analyzed according to the method of 2^–ΔΔCt^ (Livak and Schmittgen, 2001). All experiments were repeated at least three times and one representing result was shown; the primers were listed in additional information (Supplementary Table 1).

### Gut Microbiota Analysis

The 16S rRNA PCR products (V3–V4 region) obtained previously from gut microbiota were sequenced by the Biomarker Technologies company (Beijing, China) using the Illumina nova-seq 6000 platform. The gut microbiota sequencing data were summarized in Supplementary Table 2. The clean reads were clustered to obtain operational taxonomic units (OTU) by the USEARCH software based on 97.0% similarity (Edgar, 2013). Based on the Silva database (Quast et al., 2013), the feature sequences were annotated by using the Naive Bayes classifier in taxonomy, and the community composition at different levels (from phylum to species) was counted in each sample. The Quantitative Insights Into Microbial Ecology (QIIME) software was used to generate the species abundance tables at different taxonomic levels (Caporaso et al., 2010), and the R language tool was used to draw the community structure map for each sample at the taxonomic level (Charlop-Powers and Brady, 2015).

### RNA Sequencing Analysis

Each sample contained midguts from five *S. frugiperda* individuals at L6D3; each group contained three biological replicates. Total RNAs were extracted using RNAiso plus reagent (TaKaRa, Dalian, China). About 5 μg of total RNA from each sample was prepared for library construction. RNA concentration and purity were measured using NanoDrop 2000 (Thermo Fisher Scientific, Wilmington, DE, United States). RNA integrity was assessed using the RNA Nano 6000 Assay Kit of the Agilent Bioanalyzer 2100 system (Agilent Technologies, CA, United States). The VAHTS Universal V6 RNA-seq Library Prep Kit for Illumina^®^ (NR604-02, Vazyme, Nanjing, China) was used for library construction. The mRNA was enriched using the VAHTS mRNA Capture Beads (N401, Vazyme, Nanjing, China) and high-throughput sequencing was performed using Illumina nova-seq 6000 platform. The clean reads of each sample were aligned to the reference *S. frugiperda* genome (NCBI: ZJU_Sfru_1.0) by HISAT2 software (Kim et al., 2015). The aligned reads were assembled by StringTie software (Pertea et al., 2015).

The raw reads were saved in FASTQ format, and the raw sequencing data have been deposited in the NCBI Gene Expression Omnibus database under accession number: GSE175476^1^. The quality assessment was listed in the Supplementary Tables 3, 4. The Fragments Per Kilobase of transcript per Million fragments mapped (FPKM) values represent the expression levels of transcripts (Chen et al., 2020). Analysis of differentially expressed transcripts (DETs) was performed by using FPKM value of the log2(fold changes) >1 and the standard of false discovery rate (FDR) <0.001. To study the function of DETs, DETs were further classified according to Gene Ontology (GO) annotation by GOseq R packages based on Wallenius non-central hyper-geometric distribution under the standard FDR < 0.05 (Young et al., 2010). Functions of DETs pathways were determined though the Kyoto Encyclopedia of Genes and Genomes (KEGG) database, which is under the hypergeometric test and the *p*-value < 0.05 as the standard (Kanehisa et al., 2008). Gene function was also annotated based on the following databases: Nr (NCBI non-redundant protein sequences), Nt (NCBI non-redundant nucleotide sequences), Pfam (Protein family), KOG/COG (Clusters of Orthologous Groups of proteins), Swiss-Prot (A manually annotated and reviewed protein sequence database).

### Statistical Analysis

The gene expression data were analyzed by using the GraphPad Prism 5 software (GraphPad Software Inc., San Diego, CA, United States), and the significant difference was determined by the control one-way ANOVA analysis of variances and Tukey’s Multiple Comparison Test. The values are mean ± SEM (standard error of mean) (*n* = 3), and *p*-value < 0.05 indicates significant difference. The 16S rRNA sequencing and *S. frugiperda* RNA sequencing analysis have been described in the corresponding subheadings.

## Results

### Effects of Antibiotics Exposure on the *S. frugiperda* Gut Bacterial Community

**FIGURE 1:**
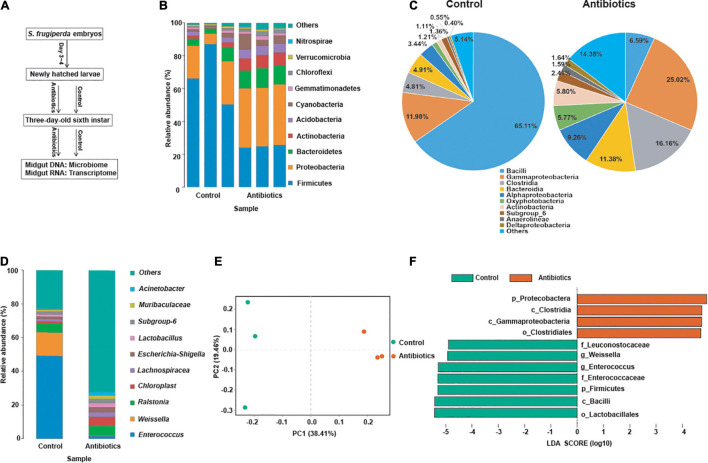
Effects of antibiotics exposure on gut microbiota structure and diversity in the *S. frugiperda* larvae. **(A)** A schematic diagram of the assay. **(B–D)** The relative abundance of gut microbiota at the phylum **(B)**, class **(C)**, and genus **(D)** levels, respectively, from the control and the antibiotics groups. **(E)** Beta-diversity was displayed by principal coordinates analysis (PCoA) based on the Bray–Curtis method in control and antibiotics groups. **(F)** Differentially abundant bacteria between the control and antibiotics groups were analyzed by LEFSe analysis with different haze levels. Linear discriminant analysis (LDA) scores indicate the degree of consistent difference in relative abundance between the control and antibiotics groups, log LDA score of >4.5 was used for the analysis.

To investigate the effect of antibiotics exposure on the gut microbial community, we reared newly hatched larvae on the artificial diet containing antibiotics mixtures (the concentration of each antibiotic was 500 μg/ml). The midguts of *S. frugiperda* at L6D3 fed on artificial diet with or without antibiotics at L6D3 were collected and analyzed (Figure 1A). A total of 390,639 high-quality reads were obtained through performing 16S rRNA pyrosequencing based on the V3–V4 region by Illumina nova-seq 6000 platform; the microbiota data were summarized in different groups (Figure 1 and Supplementary Table 2).

As shown in Figure 1, at the phylum level, Firmicutes, Proteobacteria, and Bacteroidetes were the most abundant phyla in both the control and antibiotics-treated groups, but the abundance of Firmicutes was significantly decreased in the antibiotics group compared with the control group (Figure 1B). At the class level, there was a significant decrease in the abundance of Bacilli, which was 65.11% in the control group and reduced to 6.59% in the antibiotics-treated group (Figure 1C). Moreover, at the genus level, the abundance of *Enterococcus* was dramatically reduced from 49.35% in the control group to 1.66% in the antibiotics-treated group, and the abundance of *Weissella* was also reduced from 13.85 to 0.11% after antibiotics exposure (Figure 1D). According to principal coordinates analysis (PCoA) based on the Bray–Curtis method, the result suggested that the gut microbiota of the antibiotics group deviated from that of the control group (Figure 1E). In addition, the linear discriminant analysis effect size (LEFSe) analysis was performed, and the results showed that Firmicutes (phylum), Bacilli (class), Lactobacillales (order), Enterococcaceae and Leuconostocaceae (family), and *Weissella* and *Enterococcus* (genera) were rich in the control group (Figure 1E). While Firmicutes (phylum) was not detected in the antibiotics group, Proteobacteria (phylum), Clostridia and Gammaproteobacteria (class), and Clostridiales (order) were rich after antibiotics exposure (Figure 1F). These results indicated that gut bacterial community was different after antibiotics exposure compared to the control group.

### RNA-Sequencing and Statistics of Gene Expression

To determine the effect of microbiota on gene expression in *S. frugiperda* gut, RNA samples of the midguts from control and antibiotics groups (*n* = 3) were collected and sequenced. In total, more than 4.4 million raw reads were obtained from every library with GC content and Q30 at 45 and 94%, respectively (Supplementary Table 3). Among these clean reads, 74.37–78.31% from each transcriptome were mapped to the *S. frugiperda* genome (Supplementary Table 4).

**FIGURE 2:**
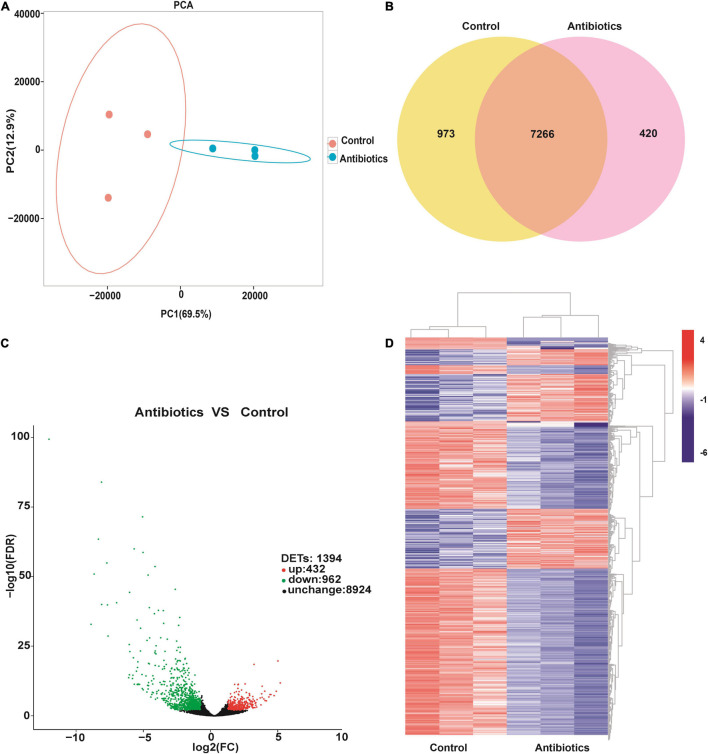
Differentially expressed transcripts in the gut of *S. frugiperda* at L6D3 between the control and antibiotics groups. **(A)** Principal component analysis (PCA) of differentially expressed transcripts based on the FPKM values of transcripts between the control and antibiotics exposure groups. **(B)** Venn diagram indicating the numbers of transcripts in the gut of *S. frugiperda* between the control and antibiotics exposure groups. **(C)** Volcano plot of differentially expressed transcripts in the gut of *S. frugiperda* between the control and antibiotics exposure groups. The red dots show the upregulated DETs, the green dots show the downregulated DETs, and the black dots show the unchanged DETs. **(D)** Hierarchical cluster heatmap showing the expression patterns of differentially expressed transcripts between the control and antibiotics group among every samples.

Based on the FPKM, principal components analysis (PCA) of transcriptome data between the control and antibiotics groups were performed, and the results indicated that PC1 principal component contributes 69.5% to the two-dimensional principal component axis; the control and antibiotics groups were significantly divided into two groups on the main component axis of PC1 (Figure 2A). To analyze the DETs between the control and antibiotics groups, the Venn figure suggested that 1,394 transcripts were differentially expressed between the two groups, 420 DETs were expressed only in the antibiotics group, 973 DETs were expressed only in the control group, and 7,266 transcripts were co-expressed in both groups (Figure 2B). Among these DETs, 432 transcripts were upregulated and 962 transcripts were downregulated in the antibiotics group vs. control group (Figure 2C). To analyze the expression pattern of DETs between the control and antibiotics groups, we drew the heatmap according to the FPKM of gene expression (Figure 2D), and the results showed that the expression levels of transcripts were significantly different in the antibiotics group compared with the control group.

### Gene Ontology Analysis of Differentially Expressed Transcripts Between the Control and Antibiotics Groups

**FIGURE 3:**
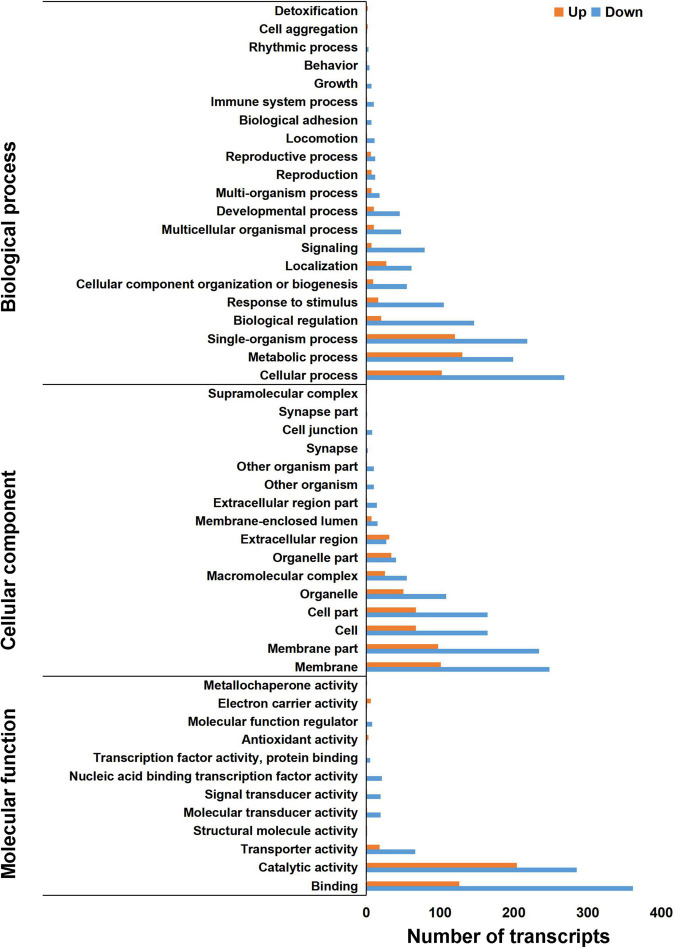
Gene Ontology annotation of differentially expressed transcripts in the gut of *S. frugiperda* at L6D3 between the antibiotics-treated and control groups at biological process, cellular component, and molecular function levels.

To further analyze the function of DETs between the control and antibiotics groups, GO analysis was performed by the GOseq R packages based on Wallenius non-central hyper-geometric distribution (Young et al., 2010). Between the antibiotics-treated and the control groups, a total of 1,394 DETs were divided into three classes: biological processes, cellular component, and molecular function (Figure 3), revealing that a large number of DETs were enriched in the GO terms including signaling, cellular component organization or biogenesis, response to stimulus, biological regulation, single-organism process, metabolic process, cellular process of biological process, membrane, membrane part, cell, cell part, organelle of cellular component, binding, catalytic activity, transporter activity, molecular transducer activity, signal transducer activity, and nucleic acid binding transcription factor activity of molecular function, and the downregulated genes were more than upregulated DETs (Figure 3). A small number of upregulated DETs were enriched in the GO term including the detoxification, supramolecular complex, extracellular region, electron carrier activity, metallochaperone activity, structural molecule activity, and antioxidant activity. Therefore, GO classification analysis suggested that bio-regulatory process and metabolic levels may be active in the midgut of *S. frugiperda* after antibiotics exposure.

### Kyoto Encyclopedia of Genes and Genomes Pathway Analysis of Differentially Expressed Transcripts Between the Control and Antibiotics Groups

**FIGURE 4:**
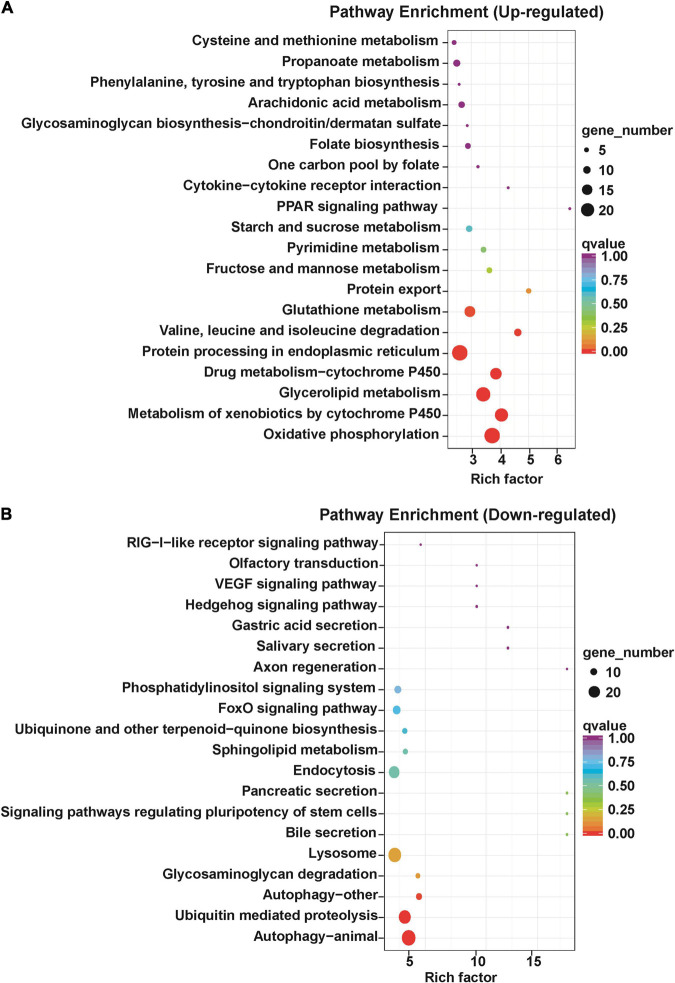
Kyoto Encyclopedia of Genes and Genomes annotation of differentially expressed transcripts in the gut of *S. frugiperda* at L6D3 after feeding antibiotics mixtures. **(A)** Top 20 of upregulated pathway enrichment. **(B)** Top 20 of downregulated pathway enrichment.

To study the signal pathway enrichment of DETs after antibiotics exposure, KEGG analysis was performed with KOBAS software according to the signal pathway data online^2^ (Mao et al., 2005; Kanehisa et al., 2008). The results revealed that differentially upregulated transcripts were enriched in oxidative phosphorylation, metabolism of xenobiotics by cytochrome P450, glycerolipid metabolism, drug metabolism-cytochrome P450, protein processing in endoplasmic reticulum, valine, leucine and isoleucine degradation and glutathione metabolism pathways (Figure 4A), implying that the energy metabolism was active after antibiotics exposure in *S. frugiperda* midgut. The differentially downregulated transcripts were mainly enriched in the autophagy, ubiquitin-mediated proteolysis, glycosaminoglycan degradation, and lysosome pathways (Figure 4B).

**FIGURE 5:**
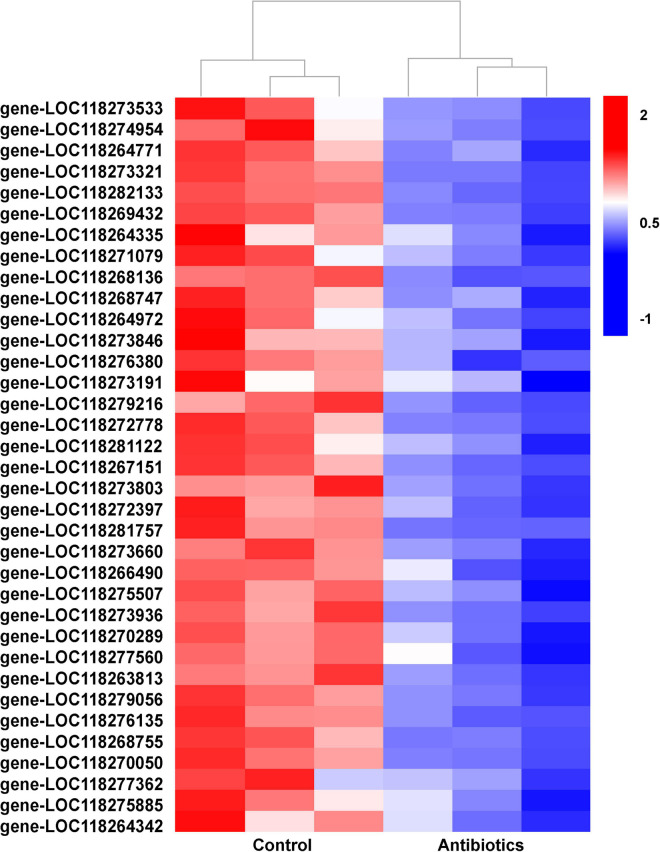
Hierarchical cluster heatmap showing the expression patterns of differentially expressed transcripts related to autophagy between the control and antibiotics groups among every samples. The values of log2 (fold change) were for the antibiotics group vs. control group and the description of differentially expressed transcripts were listed (Supplementary Table 5).

In particular, a large number of DETs related to autophagy were downregulated in the antibiotics group (Figure 5 and Supplementary Table 5). As is known, autophagy is a very important and conserved self-digestion pathway in maintaining cell homeostasis and function, and can eventually induce programmed cell death (He et al., 2017; Thorburn, 2018). In this study, autophagy-related genes were downregulated in the antibiotics-treated group; this implies that decreased autophagy may contribute to the disorder of gut microbiota to maintain intestinal balance.

### Expression of Selected Differentially Expressed Transcripts by Quantitative Real-Time PCR

**FIGURE 6:**
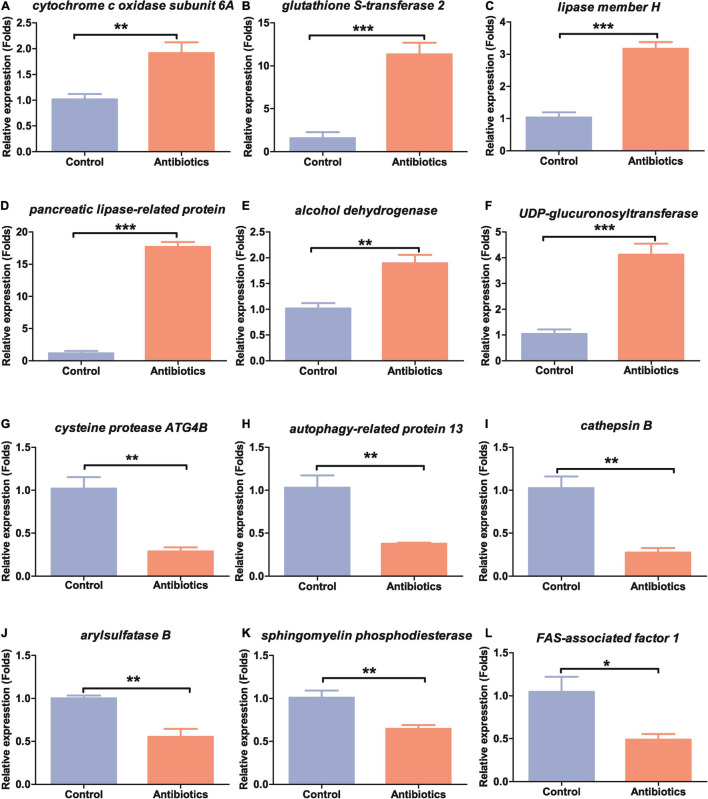
Expression patterns of the selected 12 differentially expressed genes in the guts of *S. frugiperda* between the control and antibiotics exposure groups. **(A)** *cytochrome c oxidase subunit 6A*, gene-LOC118269158; **(B)** *glutathione S-transferase 2*, gene-LOC118271637; **(C)** *lipase member H*, gene-LOC118273368; **(D)** *pancreatic lipase-related protein*, gene-LOC118273886; **(E)** *alcohol dehydrogenase*, gene-LOC118272357; **(F)** *UDP-glucuronosyltransferase 2C1*, gene-LOC118277905; **(G)** *cysteine protease ATG4B*, gene-LOC11827332; **(H)** *autophagy-related protein 13*, gene-LOC118279216; **(J)** *arylsulfatase B*, gene-LOC118268623; **(I)** *cathepsin B*, gene-LOC118264342; **(K)** *sphingomyelin phosphodiesterase*, gene-LOC118270137; **(L)** *FAS-associated factor 1*, gene-LOC118265774. *p < 0.05, **p < 0.01, and ***p < 0.001.

To validate the results of RNA-seq data, we selected 12 DETs for qRT-PCR. Of these selected DETs, six were upregulated genes, and the other six were downregulated genes in the midguts after antibiotics treatment (Table 1). These DETs were related to metabolism, oxidative phosphorylation and detoxication, and immunity reactions, which can be activated by the stimulus of the environment in insects. *Cytochrome c oxidase subunit 6A* (gene-LOC118269158) is involved in energy production and conversion; *glutathione S-transferase 2* (gene-LOC118271637), *lipase member H*, *pancreatic lipase-related protein* (gene-LOC118273886), and *alcohol dehydrogenase* (gene-LOC118272357) are the main enzymes involved in amino acid and lipid metabolism; and *UDP-glucuronosyltransferase 2C1* (gene-LOC118277905) is involved in carbohydrate transport and metabolism. All the above genes were significantly upregulated in the midgut of antibiotics group compared to the control group (Table 1 and Figure 6).

**TABLE 1:** Differentially expressed genes confirmed by qRT-PCR.

**#ID**	**GenBank ID**	**log_2_FC**	**FDR**	**Description (Blast Swiss-Prot)**
gene-LOC118269158	XM_035584112.1	2.030704248	0.000814244	Cytochrome c oxidase subunit 6A
gene-LOC118271637	XM_035587743.1	3.766858567	3.21E–06	Glutathione S-transferase 2
gene-LOC118273368	XM_035590307.1	1.412338440	0.000747025	Lipase member H
gene-LOC118273886	XM_035591062.1	4.73070998	1.97E–20	Pancreatic lipase-related protein
gene-LOC118272357	XM_035588794.1	1.768357384	0.000791343	Alcohol dehydrogenase
gene-LOC118277905	XM_035596918.1	2.198889192	0.000395065	UDP-glucuronosyltransferase 2C1
gene-LOC118273321	XM_035590222.1	–2.134623929	1.70E–14	Cysteine protease ATG4B
gene-LOC118279216	XM_035598799.1	–1.510150707	4.95E-06	Autophagy-related protein 13
gene-LOC118264342	XM_035576818.1	–2.036473832	0.000887627	Cathepsin B
gene-LOC118268623	XM_035583176.1	–3.096475659	7.43E–05	Arylsulfatase B
gene-LOC118270137	XM_035585613.1	–3.951362331	5.87E–12	Sphingomyelin phosphodiesterase
gene-LOC118265774	XM_035578938.1	–1.978805946	4.36E–06	FAS-associated factor 1

*The positive and negative value of log_2_FC indicates upregulation and downregulation, respectively.*

Many genes were also shown to be downregulated in qRT-PCR experiments as in RNA-seq data. *Cysteine protease ATG4B* (gene-LOC118273321), *autophagy-related protein 13* (gene-LOC118279216), and *cathepsin B* (gene-LOC118264342) were significantly downregulated (Table 1 and Figures 6G–I). *Arylsulfatase B* (gene-LOC118268623), a gene related to inorganic ion transport and metabolism, and *sphingomyelin phosphodiesterase* (gene-LOC118270137), a gene related to lipid transport and metabolism, were downregulated (Table 1 and Figures 6J,K). Genes related to immunity such as *FAS-associated factor 1* (gene-LOC118265774) were also downregulated (Table 1 and Figure 6L). These results suggested that *S. frugiperda* gut homeostasis was significantly affected by antibiotics exposure.

## Discussion

In this study, we exposed the newly hatched *S. frugiperda* larvae to antibiotics cocktails consisting of penicillin, gentamicin, rifampicin, and streptomycin. We firstly identified the gut bacterial community of *S. frugiperda* between the control group and antibiotics group. We found that the abundance of gut microbiota from phylum to genera level was decreased in the antibiotics group compared with the control group (Figure 1). Among the top 10 phyla, Firmicutes, Proteobacteria, Bacteroidetes, Actinobacteria, Acidobacteria, Chloroflexi, and Nitrospirae were significantly different in *S. frugiperda* midgut after antibiotics exposure, in which the dominant Firmicutes was decreased significantly and Actinobacteria was increased. Of the top genera, *Enterococcus* and *Weissella* were significantly decreased in relative abundance (Figure 1D). Therefore, antibiotics exposure affected the composition and distribution of intestinal microorganisms of *S. frugiperda.* Our results were consistent with previous studies and suggest that antibiotics could alter the microbial community in insects in lepidopteran insects (Broderick et al., 2006; Thakur et al., 2016; Raymann et al., 2017; Chen et al., 2018; Martínez-Solís et al., 2020).

The *Enterococcus* genus is known to be abundant in the intestinal tracts of various animals, possess the capacity to acquire and disseminate antimicrobial resistant determinants and the ability to produce various virulence genes that enables it to establish infections (Iweriebor et al., 2016). In many lepidopteran species, *Enterococcus* was one of most abundant genera (Paniagua Voirol et al., 2018). The *Enterococcus* genus was reduced in midgut of gypsy moth reared on antibiotic mixtures (penicillin, gentamicin, rifampicin, and streptomycin), which was necessary for *B. thuringiensis*-induced larval mortality (Broderick et al., 2006). In *Bombyx mori*, inhibition of *Enterococcus* by fluoride can increase amino acid synthesis, reduce cell carbohydrate transport and energy production (Li G. et al., 2020). Our data suggested that the abundance of *Enterococcus* was significantly reduced in the antibiotics-treated group, and the transcriptome data suggested that genes related to carbohydrate transport and metabolism and energy production were also upregulated, implying that the *Enterococcus* genus may affect the metabolism level in the gut of *S. frugiperda*. *Weissella* is a genus of lactic acid bacteria and is often found in insects (de Jonge et al., 2020; Tan et al., 2021). In *Locusta migratoria*, *Weissella* genus was the most common bacterium in the midguts and hindguts, and it would decrease after infection with the pathogens (Tan et al., 2021). It has been reported that *Weissella cibaria* strain TM128 can decrease fungal infection levels by 50% (Trias et al., 2008). In our study, the abundance of *Weissella* was also decreased in the gut of *S. frugiperda* after antibiotics treatment, implying that the resistance to pathogen infection may be decreased after antibiotics treatment.

It has been demonstrated that the gut microbiota can regulate many biological processes by influencing the related gene expression in insects (Broderick et al., 2014; Zhu et al., 2020). Therefore, we performed transcriptomic analysis by RNA-seq to further identify the genes involved in biological processes influenced by intestinal microbial population after antibiotics treatment. We found that 962 DETs were downregulated and 432 were upregulated between the control and antibiotics group (Figure 2B). GO enrichment analysis and KEGG signal pathway analysis also revealed that many metabolism processes were regulated by the DETs (Figures 4, 5). In particular, we found that *cytochrome c oxidase subunit 6A* (gene-LOC118269158) and *UDP-glucuronosyltransferase 2C1* (gene-LOC118277905) were significantly upregulated. Our results were consistent with the results in *S. litura* and Chinese bumblebee that gut microbiota affected the energy and carbohydrate metabolism after antibiotics exposure (Thakur et al., 2016; Zhang et al., 2021). Meanwhile, the *triacylglycerol lipase* gene and *glutathione S-transferase* (GST) were upregulated (Figure 6). It was speculated that gut microbiota regulated the process of hydrolyzing fats into fatty acids and glycerols by lipase. GSTs are the major enzymes in detoxification of endogenous and/or xenobiotic compounds and may also affect metabolic and signaling pathways (Ketterman et al., 2011). It was reported that *S. frugiperda* may be active in detoxifying metabolism and xenobiotic metabolism under the stimuli such as insecticides and starvation. Insects could reallocate resources when needed for survival; this makes it very difficult for pest control (Poivet et al., 2021; Shu et al., 2021; Wang et al., 2021). *Lipases* and *GSTs* were significantly increased in the gut of *S. litura* after the antibiotic (streptomycin sulfate) treatment, implying that they might play key roles in improving the efficiency of conversion of ingested food to insect biomass and further resulted in faster development, which is responsible for antibiotics tolerance (Thakur et al., 2016; Lu et al., 2021). Overall, our results were consistent with the previous studies that biological processes can be influenced by intestinal microbial population through transcriptional regulation.

It is noteworthy that genes related to the autophagy pathways were downregulated in the gut of *S. frugiperda* after antibiotics treatment according to KEGG analysis (Figures 4B, 5). In particular, the expression of *cathepsin B* (gene-LOC118264342) and *autophagy-related protein 13 homolog* (gene-LOC118279216) was significantly downregulated in the antibiotics-exposed gut of *S. frugiperda* (Figure 6). It has been suggested that cathepsin B performs its function in protein turnover as an initiator protease during lysosome-mediated autophagy/apoptosis (Bhoopathi et al., 2010; Yang et al., 2017). Moreover, it has been reported that autophagy is indispensable for intestinal homeostasis maintenance, gut ecology regulation, gut immune response, and anti-microbial protection (Larabi et al., 2020). In the *autophagy-related 5* (*Atg5*)-deficient mice model, the diversity of gut microbiota was significantly decreased (Yang et al., 2018). In our results, the abundance and diversity of dominant gut microbiota were also decreased in the gut of *S. frugiperda* after antibiotics exposure (Figure 1). Therefore, it could be speculated that intestinal autophagy of *S. frugiperda* might be suppressed with the decrease of dominant gut microbiota after antibiotics treatment; this process may play important roles in maintaining a balance of intestinal homeostasis and function during *S. frugiperda* development. The ubiquitin-mediated proteolysis pathway was downregulated in the antibiotics group compared to the control (Figure 4B). Posttranslational modification mediated by ubiquitin conjugation plays an important role in inflammation, which involved many biochemical reactions through ubiquitin systems (Hershko and Ciechanover, 1998). Some studies suggested that ubiquitin E3 ligase was associated with the gut microbiota in the inflammatory bowel diseases (Kathania et al., 2020) and immune response (Ebner et al., 2017). Therefore, ubiquitin-mediated proteolysis pathway may interact with intestinal bacteria in *S. frugiperda* gut, which could further influence the immune system, as the immune deficiency-like protein was downregulated in the antibiotics group (Figure 6). The glycosaminoglycan-degrading enzyme arylsulfatase B, a gene related to lysosomal storage disorders (Pohl et al., 2018), was also downregulated in our study. Sphingomyelin phosphodiesterase (SMPD) is a hydrolase that performs the function in metabolic reactions related to sphingomyelin (Han et al., 2018), which was downregulated in the antibiotics-exposed gut (Figure 6). These results may imply that it is necessary for *S. frugiperda* to maintain the intestinal homeostasis and metabolism to adapt to artificial diet with antibiotics mixture.

## Conclusion

Our study revealed that antibiotics mixture exposure significantly affected the gut microbiota and induced dysbiosis in *S. frugiperda*. As per our transcriptomic analysis, we further elucidate the expression of the large number of genes related to metabolism and intestinal homeostasis involved in sugar, lipid, protein metabolism, autophagy, and immunity after antibiotics exposure in the gut of *S. frugiperda.* Taken together, our findings provide fundamental information for pest control by using antibiotics to affect gut microbiota.

## Data Availability Statement

The datasets presented in this study can be found in online repositories. The names of the repository/repositories and accession number(s) can be found below: https://www.ncbi.nlm.nih.gov/, GSE175476.

## Author Contributions

YC and XYW designed the experiment and revised the manuscript. YC and HZ carried out the experiment and the bioinformatics analysis. YL and QC reared *S. frugiperda* larvae, collected and prepared RNA samples for RNA-seq, and performed some RT-PCR. X-QY provided suggestions for our study and revised the manuscript. All authors read and approved the final manuscript.

## Conflict of Interest

The authors declare that the research was conducted in the absence of any commercial or financial relationships that could be construed as a potential conflict of interest.

## Publisher’s Note

All claims expressed in this article are solely those of the authors and do not necessarily represent those of their affiliated organizations, or those of the publisher, the editors and the reviewers. Any product that may be evaluated in this article, or claim that may be made by its manufacturer, is not guaranteed or endorsed by the publisher.

## Footnotes

1
https://www.ncbi.nlm.nih.gov/geo/query/acc.cgi?acc=GSE175476

2
http://www.genome.jp/kegg/
