# Supplementary material for: Gut Microbiota Dysbiosis Influences Metabolic Homeostasis in Spodoptera frugiperda
Source: Front Microbiol. 2021 Sep 30;12:727434. doi: 10.3389/fmicb.2021.727434 (PMC8514726; doi:10.3389/fmicb.2021.727434)
Supplement: Supplementary file 1 [file Table_1.DOCX]

**Table S1** Primers for qRT-PCR**.**

| Gene name | Forward | Reverse |
| --- | --- | --- |
| *cytochrome c oxidase subunit 6A* | 5'-ATTCCCCGCAGTTGGTCTTG-3' | 5'-ATGGGAAACGCTTGGAACGA-3' |
| *glutathione S-transferase 2* | 5'-AGGTGTTTGCTGACAAGCCTC-3' | 5'-TGGTGGGCAAACTAACACTGG-3' |
| *lipase member H* | 5'-ATCCTCTCATTACATCAGCGTTCC-3' | 5'-TGTTGATGAGCCAAACCAAGAAGT-3' |
| *pancreatic lipase-related protein* | 5'-CAACCTCTGCAACCACAACC-3' | 5'-AAATGTATTGGTACATTGTCGTCCA-3' |
| *alcohol dehydrogenase* | 5'-GGAGAAGAGATCAGCACCCG-3' | 5'-TCACGGCTCTTGTAACCACC-3' |
| *UDP-glucuronosyltransferase* | 5'-GCGCTGGTGGTTTTTGGAAT-3' | 5'-ATTCGGTGGTGGGTTCTTCC-3' |
| *cysteine protease ATG4B* | 5'-CGCCGCTTCCTGATACCGT-3' | 5'-GGGTCACGGCTTTAGCAGGT-3' |
| *autophagy-related protein 13* | 5'-ACAGTAACGAGAATAACCCCCG-3' | 5'-TGGCTTCCCCACTGTAAACTCTAT-3' |
| *arylsulfatase B* | 5'-GCTTTGTAGTGAGTTCCAGGGTAG-3' | 5'-TAAGGACAAGATTCAAGCCAGACC-3' |
| *cathepsin B* | 5'-GTTGGGGAGTGGAGAACGGTAA-3' | 5'-CTCTCAATGCCACAATGGTCCT-3' |
| *sphingomyelin phosphodiesterase* | 5'-ACAAAGCCCCTACATTCAAAGTCC-3' | 5'-TGGTTTGGTTCCATACATCGTGAG-3' |
| *FAS-associated factor 1* | 5'-TCCAAAAACTCACCCACCAGAC-3' | 5'-AGTTCTACGAAGGGATTGGGAG-3' |
| *GAPDH* | 5'-CGACCAACTGTCTCGCTCCT-3' | 5'-ACAGTTTTCCAGAGGGTCCGT-3' |
