# Supplementary material for: Gut Microbiota Dysbiosis Influences Metabolic Homeostasis in Spodoptera frugiperda
Source: Front Microbiol. 2021 Sep 30;12:727434. doi: 10.3389/fmicb.2021.727434 (PMC8514726; doi:10.3389/fmicb.2021.727434)
Supplement: Supplementary file 2 [file Table_2.DOCX]

**TABLE S2** Summary of gut microbiota data in different treatments of *S. frugiperda.*

| Sample | Raw  Reads | Clean  Reads | Effective  Reads | AvgLen  (bp) | GC (%) | Q20 (%) | Q30 (%) | Effective (%) |
| --- | --- | --- | --- | --- | --- | --- | --- | --- |
| Control-1 | 44,388 | 43,708 | 42,745 | 425 | 53.92 | 98.68 | 95.0 | 96.3 |
| Control-2 | 61,564 | 60,680 | 59,857 | 427 | 52.95 | 98.71 | 95.01 | 97.23 |
| Control-3 | 44,752 | 44,129 | 42,525 | 422 | 53.22 | 98.72 | 95.08 | 95.02 |
| Antibiotics-1 | 79,937 | 79,104 | 75,596 | 417 | 54.71 | 98.91 | 95.68 | 94.57 |
| Antibiotics-2 | 80,124 | 79,336 | 75,405 | 418 | 54.83 | 98.95 | 95.8 | 94.11 |
| Antibiotics-3 | 70,735 | 69,715 | 67,644 | 416 | 54.96 | 98.73 | 95.16 | 95.63 |

Raw Reads: Original reads obtained by sequencing; Clean Reads: High-quality reads; Effective Reads: The number of effective sequences after filtering chimeras; AvgLen (bp): Average length; Q20: Percentage of every base with a Phred value of at least 20; Q30: Percentage of every base with a Phred value of at least 30; Effective (%): Percentage of Effective Reads in Raw Reads.
