# Supplementary material for: Gut Microbiota Dysbiosis Influences Metabolic Homeostasis in Spodoptera frugiperda
Source: Front Microbiol. 2021 Sep 30;12:727434. doi: 10.3389/fmicb.2021.727434 (PMC8514726; doi:10.3389/fmicb.2021.727434)
Supplement: Supplementary file 3 [file Table_3.DOCX]

**Table S3** Summary of transcriptome data of *S. frugiperda* midgut tissues*.*

| Sample | Clean Reads | Clean bases | GC (%) | Q30 (%) |
| --- | --- | --- | --- | --- |
| Control-1 | 21,735,586 | 6,511,738,022 | 45.41% | 93.49% |
| Control-2 | 20,444,873 | 6,125,200,014 | 46.01% | 93.51% |
| Control-3 | 21,614,957 | 6,459,326,826 | 44.51% | 93.93% |
| Antibiotics-1 | 22,001,082 | 6,584,467,788 | 48.02% | 93.84% |
| Antibiotics-2 | 20,111,709 | 6,017,130,250 | 47.75% | 94.42% |
| Antibiotics-3 | 21,488,588 | 6,440,484,244 | 47.03% | 93.66% |

Clean Reads: Numbers of pair-end reads in clean data; Clean bases: Total bases in clean data; GC content: Content in clean data; Q30: Percentage of every base with a Phred value of at least 30. GC: Percentage of GC bases.
