# Supplementary material for: Gut Microbiota Dysbiosis Influences Metabolic Homeostasis in Spodoptera frugiperda
Source: Front Microbiol. 2021 Sep 30;12:727434. doi: 10.3389/fmicb.2021.727434 (PMC8514726; doi:10.3389/fmicb.2021.727434)
Supplement: Supplementary file 4 [file Table_4.DOCX]

**Table S4** Statistics of clean reads mapped to *S. frugiperda* genome.

| Samples | Total  Reads | Mapped  Reads | Uniq  Mapped  Reads | Multiple  Map  Reads | Reads  Map to  “+” | Reads  Map to  “-” |
| --- | --- | --- | --- | --- | --- | --- |
| Control-1 | 43,471,172 | 32,787,891  (75.42%) | 29,233,587  (67.25%) | 3,554,304 (8.18%) | 16,052,574 (36.93%) | 16,016,802 (36.84%) |
| Control-2 | 40,889,746 | 31,673,961  (77.46%) | 25,111,587  (61.41%) | 6,562,374  (16.05%) | 15,574,188  (38.09%) | 15,494,575  (37.89%) |
| Control-3 | 43,229,914 | 32,148,447  (74.37%) | 28,510,633  (65.95%) | 3,637,814  (8.42%) | 15,700,339  (36.32%) | 15,726,925  (36.38%) |
| Antibiotics-1 | 44,002,164 | 33,707,519  (76.60%) | 28,978,805  (65.86%) | 4,728,714  (10.75%) | 16,584,801  (37.69%) | 16,404,496  (37.28%) |
| Antibiotics-2 | 40,223,418 | 31,497,543  (78.31%) | 27,259,405  (67.77%) | 4,238,138  (10.54%) | 15,457,161  (38.43%) | 15,368,985  (38.21%) |
| Antibiotics-3 | 42,977,176 | 33,374,602  (77.66%) | 29,146,495  (67.82%) | 4,228,107  (9.84%) | 16,359,291  (38.07%) | 16,311,596  (37.95%) |
