# Supplementary material for: Gut Microbiota Dysbiosis Influences Metabolic Homeostasis in Spodoptera frugiperda
Source: Front Microbiol. 2021 Sep 30;12:727434. doi: 10.3389/fmicb.2021.727434 (PMC8514726; doi:10.3389/fmicb.2021.727434)
Supplement: Supplementary file 5 [file Table_5.DOCX]

**TABLE S5** The expression pattern of differentially expressed transcripts related to autophagy between the antibiotics-treated and control group.

| #ID | log2FC | FDR | Description |
| --- | --- | --- | --- |
| gene-LOC118264342 | -1.891819661 | 3.46424E-05 | cathepsin B |
| gene-LOC118275885 | -2.688734642 | 1.35594E-09 | autophagy-related protein 2A |
| gene-LOC118277362 | -2.186543303 | 5.50667E-12 | phosphoinositide 3-kinase regulatory subunit 4 |
| gene-LOC118270050 | -3.730100081 | 1.92574E-18 | autophagy-related protein 16 |
| gene-LOC118268755 | -3.730100081 | 1.92574E-18 | serine/threonine-protein kinase |
| gene-LOC118276135 | 1.056823467 | 0.007376017 | nuclear receptor-binding factor 2 |
| gene-LOC118279056 | -2.186543303 | 5.50667E-12 | autophagy-related protein 101 |
| gene-LOC118263813 | -1.374369647 | 4.91254E-05 | transcription factor SPT20 |
| gene-LOC118277560 | -2.186543303 | 5.50667E-12 | digestive cysteine proteinase 2 |
| gene-LOC118270289 | -3.730100081 | 1.92574E-18 | lateral signaling target protein 2 |
| gene-LOC118273936 | -1.326100133 | 0.006706439 | GTPase HRas |
| gene-LOC118275507 | -2.688734642 | 1.35594E-09 | eIF-2-alpha kinase GCN2 |
| gene-LOC118266490 | -3.730100081 | 1.92574E-18 | GATOR complex protein NPRL2 |
| gene-LOC118273660 | -1.326100133 | 0.006706439 | RB1-inducible coiled-coil protein 1 |
| gene-LOC118281757 | -2.186543303 | 5.50667E-12 | uncharacterized protein LOC111357693 |
| gene-LOC118272397 | -1.326100133 | 0.006706439 | 3-phosphoinositide-dependent protein kinase 1 |
| gene-LOC118273803 | -1.326100133 | 0.006706439 | phosphatidylinositol 3-kinase catalytic subunit type 3 |
| gene-LOC118267151 | -3.730100081 | 1.92574E-18 | autophagy-related protein 9A |
| gene-LOC118281122 | -2.186543303 | 5.50667E-12 | BCL2/adenovirus E1B 19 kDa protein-interacting protein 3 |
| gene-LOC118272778 | -1.326100133 | 0.006706439 | regulator of MON1-CCZ1 complex |
| gene-LOC118279216 | -2.186543303 | 5.50667E-12 | autophagy-related protein 13 |
| gene-LOC118273191 | -1.326100133 | 0.006706439 | RB1-inducible coiled-coil protein 1 |
| gene-LOC118276380 | 1.056823467 | 0.007376017 | ras-related protein Rab-39B |
| gene-LOC118273846 | -1.326100133 | 0.006706439 | 3-phosphoinositide-dependent protein kinase 1 |
| gene-LOC118264972 | -3.730100081 | 1.92574E-18 | etoposide-induced protein |
| gene-LOC118268747 | -3.730100081 | 1.92574E-18 | serine/threonine-protein kinase |
| gene-LOC118268136 | -3.730100081 | 1.92574E-18 | protein spindle-F |
| gene-LOC118271079 | 4.051658152 | 1.59658E-07 | WD repeat domain phosphoinositide-interacting protein 4 |
| gene-LOC118264335 | -1.891819661 | 3.46424E-05 | cathepsin B |
| gene-LOC118269432 | -3.730100081 | 1.92574E-18 | autophagy-related protein 101 |
| gene-LOC118282133 | -2.186543303 | 5.50667E-12 | serine/threonine-protein kinase STK11 |
| gene-LOC118273321 | -1.326100133 | 0.006706439 | cysteine protease ATG4B |
| gene-LOC118264771 | -3.730100081 | 1.92574E-18 | WD repeat domain phosphoinositide-interacting protein 2 |
| gene-LOC118274954 | -1.326100133 | 0.006706439 | vacuole membrane protein 1 |
| gene-LOC118273533 | -1.326100133 | 0.006706439 | beclin 1-associated autophagy-related key regulator |

The positive and negative value of log_2_FC indicates up-regulation and down-regulation, respectively.
